# Giant ovarian cyst in pregnancy: a rare clinical encounter and its implications: a case report

**DOI:** 10.1093/jscr/rjaf557

**Published:** 2025-07-22

**Authors:** Josef Maria Seno Adjie, Andrew Pratama Kurniawan

**Affiliations:** Department of Obstetrics and Gynecology, Cipto Mangunkusumo Hospital, Faculty of Medicine Universitas Indonesia, Jl. Salemba Raya No. 6, Kenari, Kec. Senen, Kota Jakarta Pusat, Daerah Khusus Ibukota, Jakarta 10430, Indonesia; Department of Obstetrics and Gynecology, Cipto Mangunkusumo Hospital, Faculty of Medicine Universitas Indonesia, Jl. Salemba Raya No. 6, Kenari, Kec. Senen, Kota Jakarta Pusat, Daerah Khusus Ibukota, Jakarta 10430, Indonesia

**Keywords:** ovarian cyst, mucinosum cyst, CA-125, pregnancy, IUGR, case report

## Abstract

Giant ovarian cyst is a rare condition with <1% of ovarian cysts. A giant ovarian cyst may interfere with pregnancy and could induce some obstetrics complications and surgery challenges. This case presents a 26-year-old woman with 34 weeks of gestational age came to the polyclinic with right abdominal discomfort. Ultrasound examination showed a singleton live fetal breech presentation with the biometry of 34 weeks and a unilocular mucinous cystic mass arise from the left adnexa with low CA-125 levels. On 38 weeks of gestational age, a Cesarean section was done and evacuated a 35 cm, unilocular cyst that contained 11 L of yellowish fluid. Histopathological examination confirm the benign origin of the mass. Ultrasound examination is essential to determine the cyst characteristic and nature. When detected early in the second trimester, evacuation of the mass could have been done to prevent complications and optimizing the outcome.

## Introduction

Adnexa mass may arise from fallopian tubes or ovaries, and it is not uncommon that 20% of women may develop ovarian cysts in their lifetime [1]. Nowadays, the prevalence of ovarian cysts during pregnancy is rising, with an incidence of 0.2%–2%, since ultrasound examination is more readily available [2, 3]. Most ovarian cysts, such as follicular or corpus luteal, are small and benign. However, ovarian cysts with >10 cm are called giant ovarian cyst, and it complicates in <1% of ovarian cysts. A giant ovarian cyst may complicate pregnancy such as intrauterine fetal growth retardation (IUGR) or malpresentation [4, 5]. Thus, prompt evaluation and early diagnosis is essential in deciding management. This case study presents a pregnant woman with giant ovarian cyst.

## Case illustration

**Figure 1:**
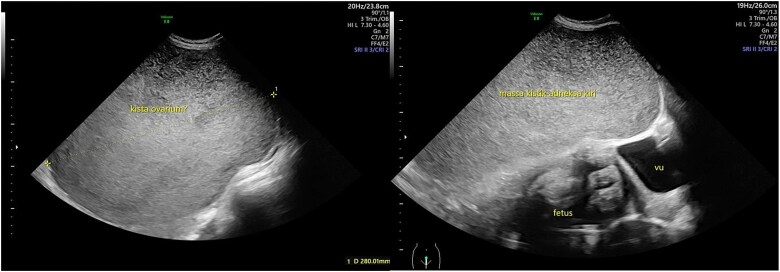
Ultrasound view of the cystic mass. The mass was unilocular without a solid part with a diameter of 28 cm.

**Figure 2:**
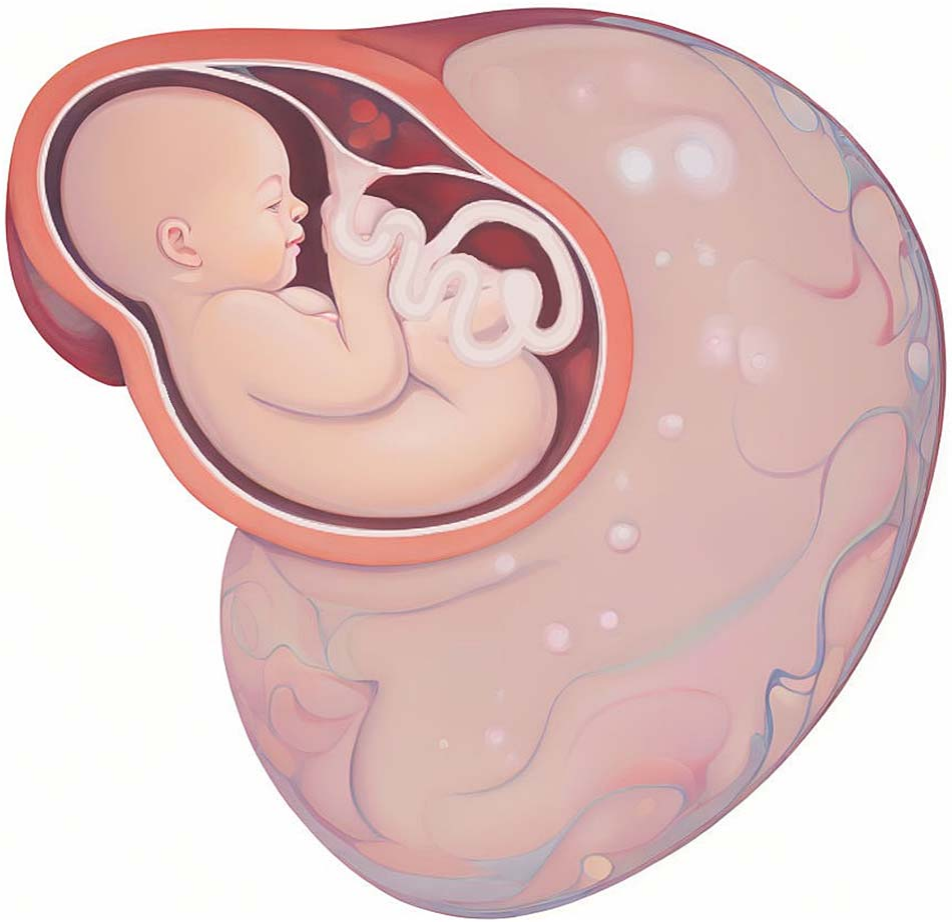
Illustration of the giant ovarian cystic mass position according to the uterus.

A 26-year-old woman with 34 weeks of gestational age, came to the polyclinic with the symptoms of right abdominal discomfort referred from another hospital with a massive ovarian cyst in pregnancy. The ovarian cyst was first detected during 28 weeks of pregnancy during her first ultrasound examination. Her abdomen was distended with a palpable mass until processus xiphoideus. Ultrasound examination showed the uterus was pushed to the right side of the abdomen with a singleton live fetal breech presentation. The fetus was around 34-weeks of gestational age, with estimated fetal weight of 2299 g, normal amniotic fluid index, and no Doppler abnormality. The ovarian cyst was unilocular from the left adnexa containing homogenous echogenocity fluid with a diameter of 28 cm, suspected of a mucinous cyst (Figs 1 and 2). A follow-up ultrasound examination showed a similar result for the cystic mass, however, the baby’s growth was declining. The Ca-125 value was below 15. Since the mass was suspected of benign origin, a Cesarean section (CS) was planned at term continued with unilateral salpingo-oophorectomy. There is no urgency to do a earlier termination of pregnancy since the fetus cardiotocography and doppler studies is within normal limit.

**Figure 3:**
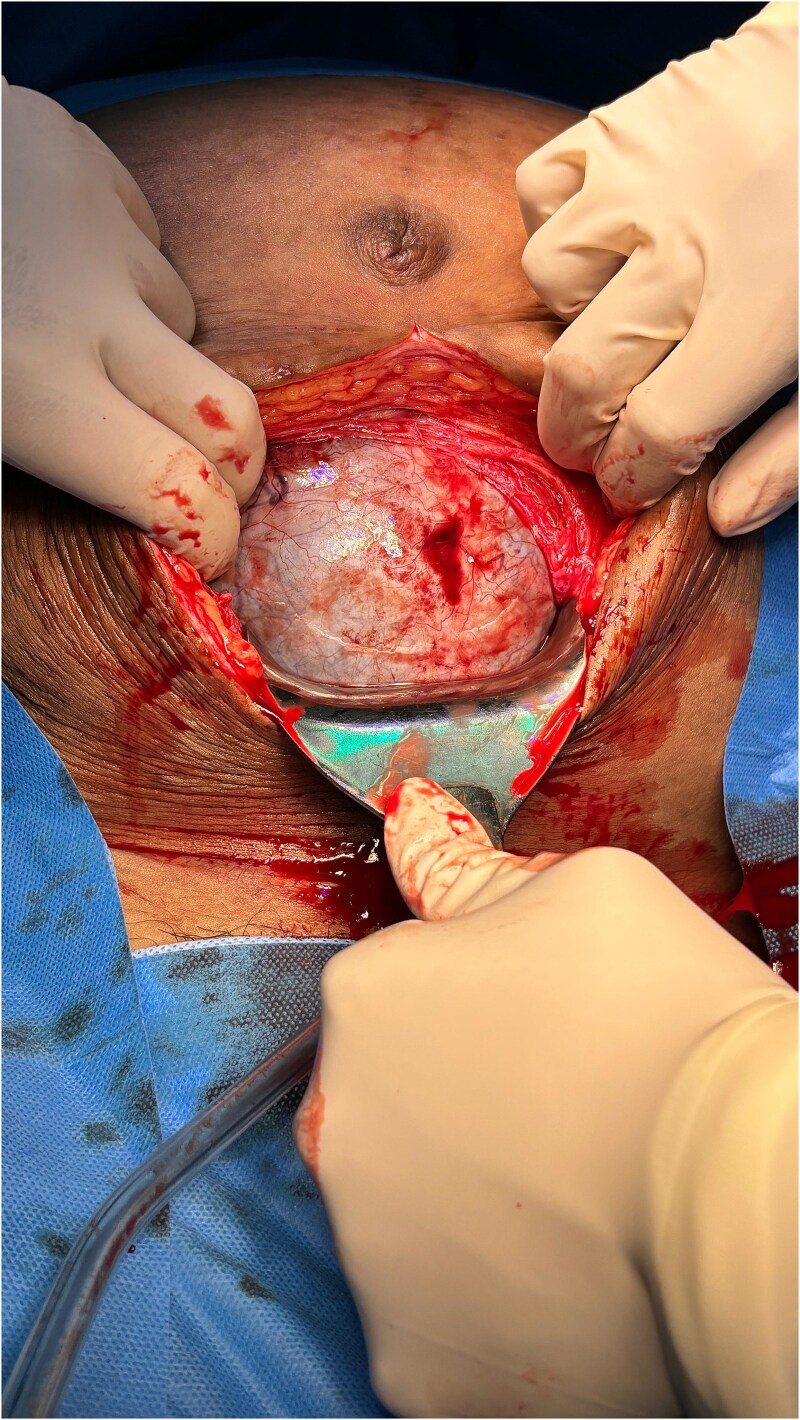
Abdominal view after the peritoneum was opened. Show large cystic mass filled the abdominal cavity obstructing the access to the uterus.

**Figure 4:**
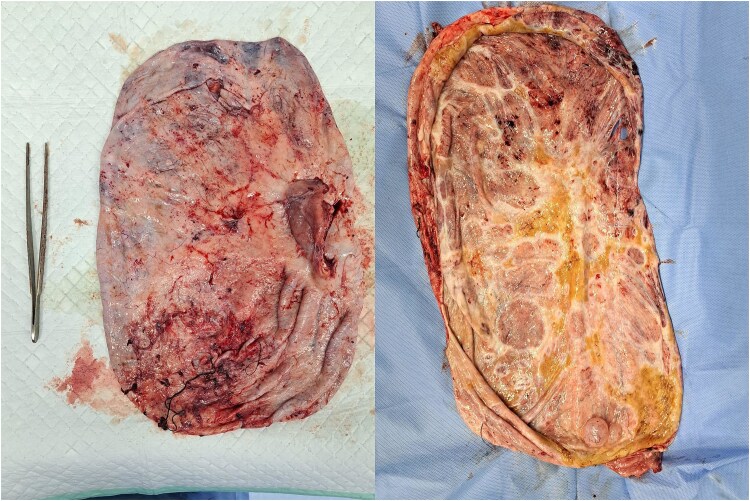
The outer and inside parts of the ovarian tumor were unilocular, with no solid part found.

The surgery was done at 38 weeks of gestational age. A median incision was done to optimize the surgery field. When the peritoneum was opened a large smooth cystic mass filled the entire abdominal cavity, a tobacco sac suture was done to decompressed the mass (Fig. 3). After 11 L of yellowish fluid, the cyst was identified coming from the left ovary, and the uterus was pushed laterally to the right. CS was performed to deliver a 2350 g boy, Apgar score of 9/10. Salpingo-oophorectomy was then performed after the CS. The operation was done without complication and transfusion of blood products. The mass was 35 cm in diameter, unilocular, without any solid part, thus showing the characteristic of benign mass (Fig. 4).

The postoperative hemoglobin level was 9.1 g/dl, and she was discharged without complications. The pathological result of the specimen showed ovarian cystadenoma mucinosum. The patient and her baby were in good condition during follow-up, and the wound was healed completely. There is no plan for further treatment or examination.

## Discussion

In this case, the ovarian cyst was found late during the early third trimester. If the patient was diagnosed sooner, an operation could be done in the second trimester to remove this ovarian cyst, preventing complications and alleviating the symptoms caused by this large abdominal mass. Thus, ultrasound examination is essential in early antenatal care. Ultrasound is also the most efficient way to determine the nature of an ovarian cyst. International Ovarian Tumor Analysis (IOTA) has provided an easy descriptor of ovarian cyst called simple rules [6]. A study found that IOTA simple rules are accurate even for non-expert examiners to diagnose the characteristics of an ovarian cyst [7]. The main characteristics that need to be explored are locule formation, cyst content, solid part of the masses or papillary structure, vascular score, shadows, and ascites. From the case, the mass was probable benign with signs of unilocular, low-echogenocity homogenous cyst content, no solid part, and no vascular score. A simple salpingo-oophorectomy could be performed to excise the mass. MRI is another imaging modality that could be done when the ultrasound result is insufficient [8]. Another laboratory modality that could be used is CA-125 levels or MUC-16, a protein that shed from the epithelial cells of the ovary. The number is expected to increase if the mass is malignant with the upper limit at 35 U/ml [9]. A literature study found that the median CA-125 levels in low-grade serous carcinoma, or mucinous carcinoma, is ~53–413 U/ml [9]. Nevertheless, CA-125 levels may arise during pregnancy, delivery (up to 48 hours), and obese women [9, 10]. In this patient, the CA-125 level was very low (< 15 U/ml), ensuring the benign origin.

Obstetric complications such as preterm labor, malpresentation, IUGR and prolonged labor due to obstructed birth passage may arise from giant ovarian cyst [11]. In this case, the compression from the giant ovarian cyst induce malpresentation in which the fetus had a breech presentation and restricting the growth of the uterus and fetus. This finding was similar to other reports that giant ovarian cysts complicate pregnancy by IUGR and malpresentation [11, 12]. On the other hand, non-obstetrical complications of the cyst were rupture, haemorrhage, obstruction of bowel or bladder, and torsion [1, 13]. Torsion is more common in a mobile cyst with a long pedicle and in a cyst >10 cm in the first trimester [14]. In this case, their mass was so large that torsion did not happen. However, there is still a risk of rupture or haemorrhage. The mass histopathology was benign, cystadenoma musinosum. Its recurrence rate was low. However, there is a risk of pseudomyxoma peritonei if the cyst is ruptured and spilled to the peritoneum [13].

## Conclusion

Giant ovarian cysts during pregnancy are rare. Ultrasound is the main modality to determine the mass origin and chance of malignancy. When the ovarian cyst was found in an early pregnancy, surgical management could be done in the second trimester to prevent complications. However, when it was identified in the third trimester, expectant management and close monitoring could be done to detect complications and time for pregnancy termination.

## Consent

The patient had given an informed consent for participation and publication of the result.
